# Enriched Environment Attenuates Ferroptosis after Cerebral Ischemia/Reperfusion Injury via the HIF-1*α*-ACSL4 Pathway

**DOI:** 10.1155/2023/5157417

**Published:** 2023-02-08

**Authors:** Jingying Liu, Qihang Luo, Jie Ke, DongDong Zhang, Yang Xu, Weijing Liao, Xiuping Chen, Xin Zhang

**Affiliations:** ^1^Department of Rehabilitation Medicine, Zhongnan Hospital of Wuhan University, Wuhan, China; ^2^Department of Neurosurgery, Renmin Hospital of Wuhan University, Wuhan, China; ^3^Department of Rehabilitation Medicine, The First Affiliated Hospital of Nanchang University, Jiangxi, China

## Abstract

Enriched environment (EE) has been proven to be an effective intervention strategy which can improve neurofunctional recovery following cerebral ischemia/reperfusion (I/R) injury. However, it still needs further investigation for the underlying mechanisms. Recently, it has been shown that ferroptosis played an essential role in the pathophysiological development of ischemic stroke (IS). This study is aimed at investigating whether EE plays a neuroprotective role by attenuating ferroptosis after cerebral I/R injury. We used middle cerebral artery occlusion/reperfusion (MCAO/R) to build a model of cerebral I/R injury. To evaluate the effect of EE on neurological recovery, we used the modified neurological severity score (mNSS) and the Morris water maze (MWM). We used the western blot to detect the protein levels of glutathione peroxidase 4 (GPX4), hypoxia-inducible factor-1*α* (HIF-1*α*), and acyl-CoA synthetase long-chain family member 4 (ACSL4). We used the quantitative real-time PCR (qRT-PCR) to measure the mRNA levels of ACSL4 and inflammatory cytokines including tumor necrosis factor alpha (TNF*α*), interleukin-6 (IL-6), and interleukin 1 beta (IL-1*β*). The occurrence of ferroptosis was detected by TdT-mediated dUTP nick-end labeling (TUNEL) assay, diaminobenzidine- (DAB-) enhanced Perls' staining, iron level assays, and malondialdehyde (MDA) level assays. The results verified that EE enhanced functional recovery and attenuated ferroptosis and neuroinflammation after cerebral I/R injury. EE increased the expression of HIF-1*α* while inhibited the expression of ACSL4. Our research indicated that EE improved functional recovery after cerebral I/R injury through attenuating ferroptosis, and this might be related to its regulation of the neuroinflammation and HIF-1*α*-ACSL4 pathway.

## 1. Introduction

Stroke is the second leading cause of death which also acts as one of the diseases with the highest disability rates worldwide [1, 2]. Ischemic stroke (IS) is the predominant type of stroke, but the treatment options for IS are very limited [3]. To alleviate the neurological deficits caused by IS, intensive poststroke rehabilitation is one of the few valid options. Rehabilitation after stroke has been shown to diminish the risks of stroke recurrence and improve functional recovery [4]. Therefore, it is vital to explore more rehabilitative strategies to alleviate the neurological deficits caused by IS.

Enriched environment (EE) is a stimulus complex. By providing the housing animals with larger space, novel play props, and more social partners, the animals housed in the EE have more sensory, cognitive, motor, and social stimulation than in the standard conditions (SC) [5, 6]. It has been proven that EE could be a bright strategy to improve cognitive-behavioral performance and functional recovery after IS [7–11]. In animal experiments, EE has been shown to reduce brain infarct volumes and enhance angiogenesis in I/R rats [9, 11]. EE also influenced cell death following IS. It promoted autophagy while inhibited pyroptosis and apoptosis of neurons in the penumbra which eventually facilitate functional recovery [10, 12, 13]. Despite the fact that multiple pathophysiological processes are involved in cerebral I/R injury and its recovery, research is needed to clarify how EE improves functional recovery after IS with the aim of clinical transformation in the future [14–16].

Ferroptosis is a recently discovered form of cell death that is distinguished from apoptosis, pyroptosis, and autophagy, characterized by the iron-dependent accumulation of lipid hydroperoxides [17]. Recent studies have shown that I/R injury can induce reactive oxygen species (ROS) and lipid peroxidation to trigger ferroptosis [18]. Following the stroke, ferroptosis occurred and the iron levels in the brain increased [19]. The inhibition of ferroptosis effectively reduced poststroke injury [20]. Inhibition of ferroptosis and promotion of the levels of glutathione peroxidase 4 (GPX4) by selenium significantly protect neurons after stroke [21]. GPX4 plays an important role in ferroptosis by inhibiting lipid peroxidation and thus regulating ferroptosis [22]. When the activity of GPX4 is inhibited, ferroptosis will be triggered as the lethal amount of lipid peroxidation accumulation [23]. Postischemic neuroinflammation might play a crucial role in ferroptosis. Inflammatory cytokines (e.g., TNF*α*, IL-1*β*, and IL-6) have been demonstrated to directly affect GPX4 levels, implying that these cytokines may regulate ferroptosis [24]. Although there is growing evidence that ferroptosis is involved in cerebral I/R injury, the relationship between ferroptosis and the neuroprotective effects of EE after IS remains unknown.

Acyl-CoA synthetase long-chain family member 4 (ACSL4) is an enzyme involved in the metabolism of polyunsaturated fatty acids (PUFA) [25]. ACSL4 could facilitate free PUFAs to synthesize PUFA-phosphatidylethanolamines, which are unstable and prone to lipid peroxidation [18]. Lipids are important components of the brain, and increased ACSL4 expression makes lipids more susceptible to peroxidation, which exacerbates the occurrence of ferroptosis [26]. Studies have shown that ACSL4 makes cells more susceptible to ferroptosis [27]. Recently, ACSL4-modulated ferroptosis has been demonstrated in cerebral and intestinal I/R injury, indicating that ACSL4 is a novel regulator of ferroptosis [27, 28]. Hypoxia-inducible factor-1*α* (HIF-1*α*) is a critical regulator in cerebral I/R injury. It modulates the expression of a set of genes involved in cellular adaptation to hypoxia [29–31]. According to recent research, HIF-1*α*, as a transcriptional factor, may bond to the ACSL4 promoter region to suppress its transcription [32]. In addition, HIF-1*α* has been shown to inhibit ACSL4 transcription in cerebral I/R injury [28]. The inhibition of HIF-1*α* could increase ROS levels, implying that HIF-1*α* may be an important target in the regulation of ferroptosis [33].

We hypothesized that EE treatment could improve neurological outcomes after cerebral I/R injury by attenuating ferroptosis. In this study, we investigated how EE affected the expression of inflammatory cytokines, ACSL4, and HIF-1*α* and the potential link between them. We also looked into whether EE attenuated ferroptosis after cerebral I/R injury, as well as the underlying molecular mechanism.

## 2. Materials and Methods

### 2.1. Animal and Subject

**Figure 1:**
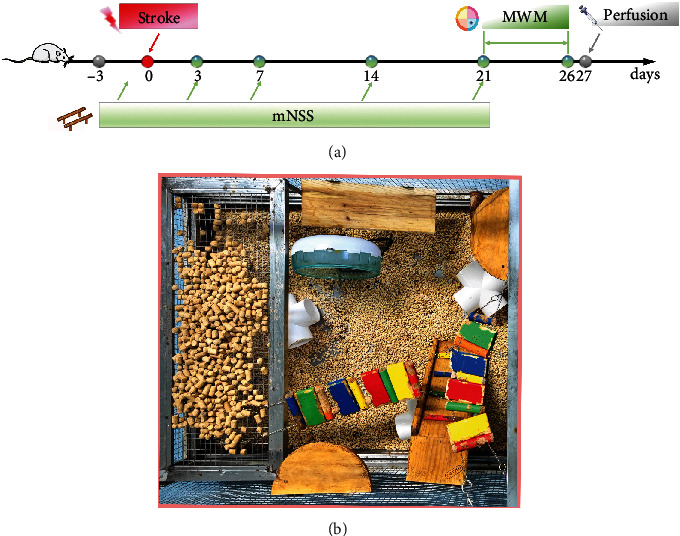
Experimental flow and enriched environment setting. (a) Timeline of the experimental procedure for this experiment. Rats were tested on day 3, 7, 14, and 21 by using the modified neurological severity score (mNSS) to assess sensorimotor deficits. On day 21 to 26, rats were tested using the Morris water maze (MWM) to assess spatial learning and memory. (b) The setting of an enriched environment in this experiment.

All animal experiments were authorized by the Animal Care and Use Committee at Wuhan University (WP2020-08052). Animal welfare was valued during the experiments, and every effort was made to mitigate the pain of experimental animals. During the experimental process, every effort was made to keep animal mortality and suffering to a minimum. Specific pathogen-free male SD rats (6–7 weeks old, 210–220 g) were purchased from Beijing Vital River Laboratory Animal Technology Company. Rats were housed in an artificially controlled environment (55 ± 5% relative humidity, 20 ± 2°C, and photoperiod from 8:00 to 20:00) and had unrestricted access to food and water. Figures 1(a) and 1(b) illustrate the setting of EE and the experimental timeline. After three days of adaption training, the rats were marked and randomized into four groups as follows: (1) SSC group: the sham+standard condition group, (2) SEE group: the sham+enriched environment group, (3) ISC group: the ischemia/reperfusion+standard condition group, and (4) IEE group: the ischemia/reperfusion+enriched environment group.

### 2.2. Rat Middle Cerebral Artery Occlusion and Reperfusion (MCAO/R) Model

As previously reported, middle cerebral artery occlusion and reperfusion (MCAO/R) was used to build the cerebral I/R injury model in rats [13, 34]. Briefly, isoflurane was used to anesthetize rats with a face mask (induction concentration: 3%, maintenance concentration: 1.5%, in 2 : 1 N_2_O: O_2_). A midline incision of approximately 2 cm is made in the neck and the left common carotid artery (CCA), and the left external carotid artery and left internal carotid artery (ICA) are separated. A small incision was made in the CCA, and a 5-0 nylon monofilament (Beijing Xinong Biotech, China) was passed through the incision into the ICA approximately 20 ± 2 mm. The nylon monofilament wire was carefully removed 90 minutes later to ensure the reperfusion [35]. The sham-operated rats were operated on the same as the surgical group except that the nylon monofilament was not inserted. After surgery, the rats were scored on a five-point neurological deficit using a blinded method (only scores 1-3 were retained and excluded scores 0 and 4) [36].

### 2.3. Housing Conditions

The rats were placed in the respective environment 24 hours following MCAO/R according to the groupings. The following are the specifics of standard condition (SC): three rats were housed in a cage (40 cm × 30 cm × 20 cm) with bedding, food, and water. The specifics of the enriched environment (EE) are as follows: six rats were kept in a special cage (90 cm × 75 cm × 50 cm) with stairways, stages, swing boards, tunnels, and running wheels. To ensure novelty and exploration, the cage settings were changed every three days (Figure 1(b)).

### 2.4. Behavioral Tests

The modified neurological severity score (mNSS) was used to evaluate sensorimotor deficits (including motor, sensory, reflex, and balance assessments), and higher scores indicated more severe neurological damage (*n* = 6/group) [12]. The Morris water maze (MWM) test was used to assess the spatial learning and memory abilities of rats (*n* = 6/group) [37]. For the first five days, the platform position was fixed and each animal was trained 4 times a day, each time the rats were placed in the water from four different quadrants, and their movement trajectory and time were recorded. The rats were permitted to rest on the platform for 15 seconds after reaching it in less than 60 seconds. If it took more than 60 seconds to reach the platform, the tester would lead it to stay at the platform for 15 seconds. We recorded the escape latency of each animal. On day 6, the platform was withdrawn and rats were permitted to swim freely for 1 min. An Animal Video Tracking Analysis System was used to record swimming trajectories, time in correct quadrant, and platform crossovers to make a reasonable judgment on the learning memory ability of the spatial location (Anilab Scientific Instruments Co., Ltd., China).

### 2.5. Western Blot

Protein sample tissues were collected from the peri-infarct cortex. Proteins were separated on SDS/PAGE gels (10%) and electroblotted into a PVDF membrane. The PVDF membranes were then incubated in 5% nonfat milk at room temperature for 1 h and then incubated with primary antibodies overnight at 4°C. The membrane was washed three times with phosphate-buffered saline (PBS) containing 0.1% Tween-20 and incubated in the secondary antibodies (ABclonal, China) for 1 h at room temperature. We used the Bio-Rad system to scan the proteins (*n* = 3/group). The primary antibodies used in this experiment were listed as follows: GAPDH (Proteintech, China), GPX4 (Abmart, China), HIF-1*α* (Zen-Bio, China), and ACSL4 (Abcam, UK).

### 2.6. Quantitative Real-Time PCR (qRT-PCR) Analysis

According to the manufacturer's instructions, total RNA was extracted from peri-infarct cortex tissues using TRIzol reagent (Invitrogen, USA). We performed qRT-PCR to detect mRNA levels by using SYBR Premix Ex Taq II (Takara, Japan) in a 2.1 Real-Time PCR System (Bio-Rad, USA) according to the manufacturer's protocol. The relative Ct method was used for data comparison, and GAPDH was set as an internal control (*n* = 3/group). The primer sequences are given below.

IL-1*β* (F): TGACTTCACCATGGAACCCG

IL-1*β* (R): TCCTGGGGAAGGCATTAGGA

IL-6 (F): TCCTACCCCAACTTCCAATGCTC

IL-6 (R): TTGGATGGTCTTGGTCCTTAGCC

TNF-*α* (F): TTGCTTCTTCCCTGTTCC

TNF-*α* (R): CTGGGCAGCGTTTATTCT

ACSL4 (F):TATGGGCTGACAGAATCATG

ACSL4 (R): CAACTCTTCCAGTAGTGTAG

GAPDH (F): CGCTAACATCAAATGGGGTG

GAPDH (R): TTGCTGACAATCTTGAGGGAG

### 2.7. Immunofluorescence Assays

Paraffin sections were obtained from brain specimens of different groups. They were hydrated and antigen retrieval was performed using a buffered solution with sodium citrate. Sections were blocked with 5% bovine serum albumin for 1 h. And the sections were then incubated with primary antibody for 12 h at 4°C. PBS buffer was used to wash sections 3 times (5 min each), and sections were incubated with fluorescent secondary antibody for 1 h at room temperature. Nuclei were stained with DAPI (Antgene, China). The images were observed and taken by a BX53 microscope (Olympus, Japan). The number of positive cells was calculated using ImageJ (*n* = 3/group).

### 2.8. TdT-Mediated dUTP Nick-End Labeling (TUNEL) Assay

TUNEL assay is a well-established, fast, and simple technique to detect and quantify neurons undergoing regulated cell death [38]. TUNEL staining was performed according to the method described by the manufacturer. Briefly, paraffin sections were pretreated as previously described. The sections were then reacted with proteinase K for 30 min at room temperature. PBS buffer was used for sections' washing (5 min each for 5 times) followed by incubating with the TUNEL assay solution. Then, the reaction was stopped and the slides were washed three times with PBS. Nuclei were stained with DAPI (Antgene, China). The images of the peri-infarct cortex were observed and taken by a BX53 microscope (Olympus, Japan), and the number of positive cells was calculated using ImageJ (*n* = 3/group).

### 2.9. Diaminobenzidine- (DAB-) Enhanced Perls' Staining

Prussian Blue Iron Stain Kit (Solarbio, China) was used to detect iron deposits in rat brain tissue (*n* = 3/group). Paraffin sections were pretreated as previously described and washed with distilled water. 3% hydrogen peroxide was used to block paraffin sections for 10 minutes. Perls' staining solution was configured according to the instructions. Then, the sections were put into the Perls' staining solution and incubated at 37°C for 12 hours. After washing 3 times (5 min each) with PBS, sections were stained with DAB Horseradish Peroxidase Color Development Kit (Beyotime, China). The images were observed and taken by a BX53 microscope (Olympus, Japan).

### 2.10. Iron and Malondialdehyde (MDA) Level Assays

The level of iron in rat brains was measured using the tissue iron assay kit (Nanjing Jiancheng Bio, China) according to the manufacturer's instructions. The concentration of lipid peroxidation product malonaldehyde (MDA) in rat brains was measured by an MDA assay kit (Beyotime, China) following the manufacturer's instructions (*n* = 3/group).

### 2.11. Statistical Analysis

The nonparametric Kruskal-Wallis test was used to analyze the mNSS data. MWM data were analyzed by Tukey's *post hoc* test and two-way repeated measures ANOVA. One-way ANOVA and Tukey's *post hoc* test were used to compare differences between groups. We used SPSS 23 software and GraphPad Prism 8 for statistical analysis. All data were expressed as mean ± standard deviation (SD). Statistical significance was established as *p* < 0.05.

## 3. Results

### 3.1. Enriched Environment Attenuated Neurological Deficits Caused by Cerebral I/R Injury

**Figure 2:**
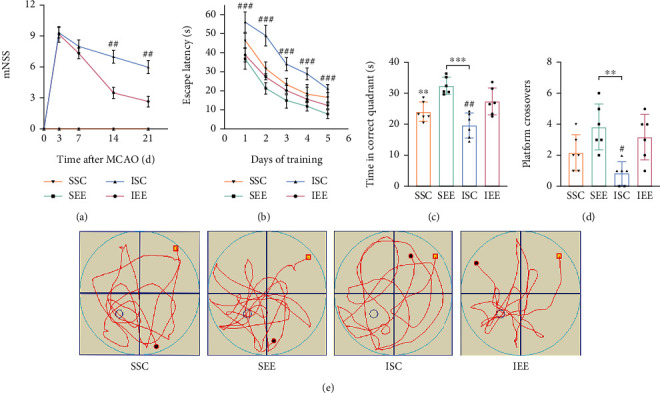
Enriched environment attenuated neurological deficits caused by I/R injury. (a) Changes in mNSS for different groups on day 3, 7, and 21. Rats were trained preoperatively to avoid errors. (b) The escape latency in the spatial learning phase. (c, d) Time in the correct quadrant and the crossovers in the target quadrant was recorded and analyzed. (e) Representative swimming trajectories of SSC, SEE, ISC, and IEE groups in the probe trials. *n* = 6. Data are expressed as mean ± SD. ^∗∗^*p* < 0.01 and ^∗∗∗^*p* < 0.001 vs. SEE group; ^#^*p* < 0.05 and ^##^*p* < 0.01 vs. IEE group.

Cerebral I/R injury caused significant behavioral dysfunction. Various behavioral tests were performed to determine whether EE could attenuate neurological deficits caused by cerebral I/R injury. Neurological deficits were assessed in rats using mNSS at 3, 7, 14, and 21 days after cerebral I/R injury. EE significantly reduced neurological deficits despite the persistence of sensorimotor impairment because of cerebral I/R injury (Figure 2(a); *p* < 0.01). At 21-26 days after cerebral I/R injury, the MWM was used to assess the spatial learning and memory ability of rats. During the spatial learning phase, the escape latency was decreasing with the increasing training time. During the first five days of training, rats in the I/R groups (ISC and IEE) took more time to reach the platform compared with the sham-operated groups (SSC and SEE). However, rats in the IEE group exhibited shorter escape latency compared with the ISC group (Figure 2(b); *p* < 0.001). At the end of the five-day spatial learning training, probe trials were conducted. In the I/R groups, the IEE group performed better than the ISC group, as they stayed longer in the correct quadrant and revealed more crossovers(Figures 2(c)–2(e); *p* < 0.01 and *p* < 0.05). These results demonstrated that neurological deficits could be reduced by EE following I/R injury.

### 3.2. Enriched Environment Reduced Ferroptosis after Cerebral I/R Injury

**Figure 3:**
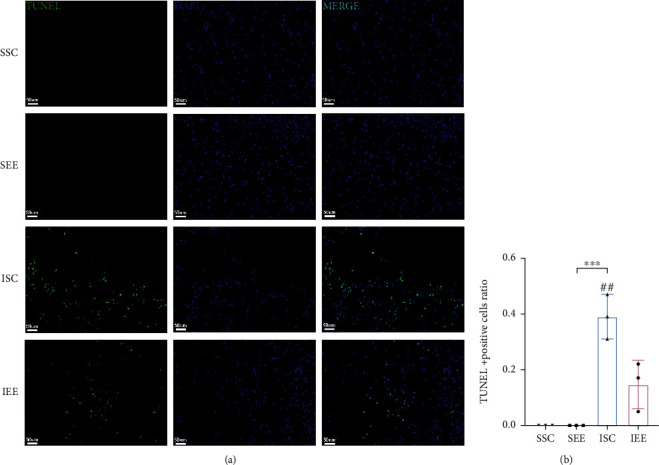
Enriched environment reduced regulated cell death in the peri-infarct cortex. (a) TUNEL staining for evaluation of the regulated cell death in the peri-infarct cortex. (b) Quantitative analysis for the number of TUNEL-positive cells. Scale bars, 50 *μ*m. *n* = 3. Data are expressed as mean ± SD. ^∗∗∗^*p* < 0.001 vs. SEE group; ^##^*p* < 0.01 vs. IEE group.

**Figure 4:**
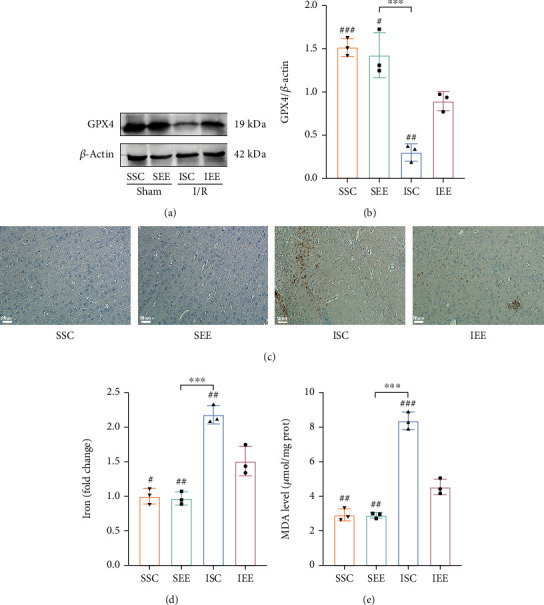
Enriched environment reduced iron deposition and ferroptosis. (a, b) Western blots and quantification of GPX4 in the peri-infarct cortex. (c) Perls' staining of iron in the peri-infarct cortex. (d) Quantitative analysis of iron levels in the peri-infarct cortex. (e) Quantitative analysis of MDA levels in the peri-infarct cortex. Scale bars, 50 *μ*m. *n* = 3. Data are expressed as mean ± SD. ^∗∗∗^*p* < 0.001 vs. SEE group; ^#^*p* < 0.05, ^##^*p* < 0.01, and ^###^*p* < 0.001 vs. IEE group.

To investigate whether EE enhanced functional recovery by inhibiting ferroptosis after cerebral I/R injury, we used TUNEL staining, DAB-enhanced Perls' staining, iron assay kit, and MDA assay kit to detect ferroptosis-related features. We used TUNEL staining to detect whether EE reduced the occurrence of regulated cell death in the peri-infarct cortex. Massive positive cells were observed in the I/R groups, whereas few positive cells were detected in the sham-operated groups. EE significantly reduced the number of positive cells, and the proportion of positive cells in the IEE group was obviously lower than in the ISC group (Figures 3(a) and 3(b); *p* < 0.01). These results demonstrated that EE inhibited regulated cell death. The western blot results of GPX4 showed that I/R injury significantly decreased the expression level of GPX4, while EE significantly improved the level of GPX4 after I/R injury (Figures 4(a) and 4(b); *p* < 0.01 and *p* < 0.001). In the I/R groups, Perls' staining showed that the number of iron depositions in the peri-infarct cortex was significantly increased in comparison with the sham-operated groups (Figure 4(c)). The iron assay kit was used to further explore iron alterations, and the results showed that iron levels elevated following I/R injury. However, EE could reduce the iron deposition caused by cerebral I/R injury (Figure 4(d); *p* < 0.01). Significant accumulation of lipid peroxides is a typical feature that distinguishes ferroptosis from other types of regulated cell death [39]. Therefore, we applied the MDA assay to assess lipid peroxidation alterations in the peri-infarct cortex. The results showed that I/R injury significantly elevated MDA levels, while the IEE group showed a significant decrease in MDA levels compared to the ISC group (Figure 4(e); *p* < 0.001). Collectively, all of these data implicated that ferroptosis after cerebral I/R injury was suppressed by EE.

### 3.3. Enriched Environment Reduced the Expression of the Inflammatory Cytokines

**Figure 5:**
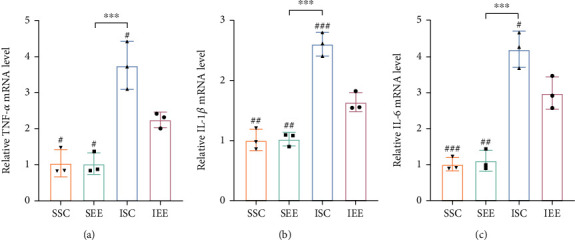
Enriched environment reduced the expression of the inflammatory cytokines. (a) Quantitative analysis of TNF-*α* mRNA levels in the peri-infarct cortex. (b) Quantitative analysis of IL-1*β* mRNA levels in the peri-infarct cortex. (c) Quantitative analysis of IL-6 mRNA levels in the peri-infarct cortex. *n* = 3. Data are expressed as mean ± SD. ^∗∗∗^*p* < 0.001 vs. SEE group; ^#^*p* < 0.05, ^##^*p* < 0.01, and ^###^*p* < 0.001 vs. IEE group.

Considering that ferroptosis might be regulated by inflammatory cytokines. The expression levels of inflammatory factors (TNF-*α*, IL-1*β*, and IL-6) were measured by qRT-PCR. The results revealed that the mRNA levels of inflammatory factors were increased in the I/R groups compared with the sham-operated groups. However, the mRNA levels of inflammatory factors were obviously less in the IEE group in comparison with the ISC group (Figures 5(a)–5(c); *p* < 0.05, *p* < 0.001, and *p* < 0.05).

### 3.4. Enriched Environment Reduced the Expression of ACSL4 after Cerebral I/R Injury

**Figure 6:**
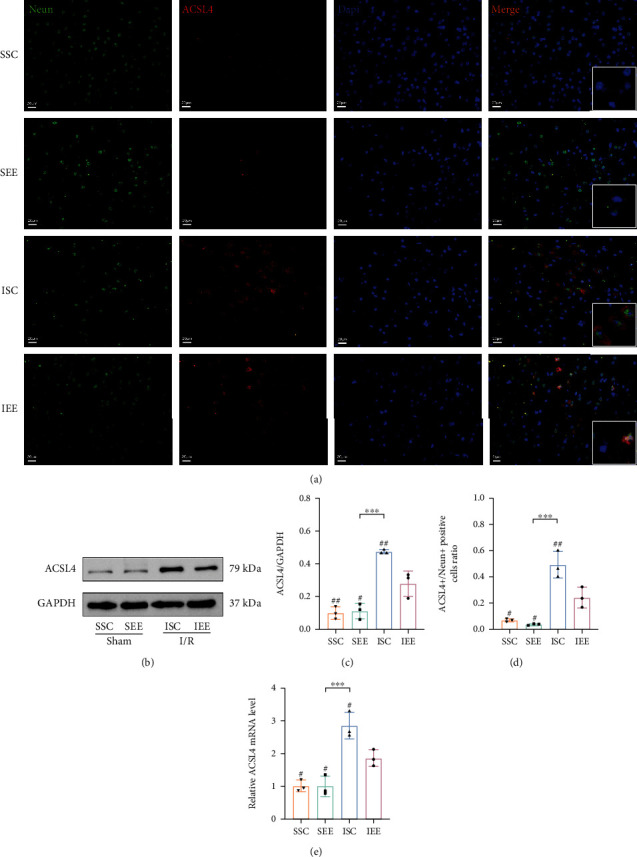
Enriched environment reduced the expression of ACSL4 after I/R injury. (a, d) Double immunostaining of Neun and ACSL4. Statistical analysis of the positive rate was shown. (b, c) Western blots and quantification of ACSL4 in the peri-infarct cortex. (e) Quantitative analysis of ACSL4 mRNA levels in the peri-infarct cortex. Scale bars, 20 *μ*m. *n* = 3. Data are expressed as mean ± SD. ^∗∗∗^*p* < 0.001 vs. SEE group; ^#^*p* < 0.05 and ^##^*p* < 0.01 vs. IEE group.

ACSL4 expression was positively associated with the occurrence of ferroptosis [40], and exploring the effect of EE on ACSL4 expression would help to investigate the role of EE on ferroptosis. As shown in the western blot results, the expression of ACSL4 was obviously increased following cerebral I/R injury. However, EE reduced the expression of ACSL4 (Figures 6(b) and 6(c); *p* < 0.01). The immunofluorescence results showed that the ACSL4 was increased in neurons after I/R injury and the proportion of ACSL4/Neun-positive cells in the ISC group was obviously higher compared with the IEE group (Figures 6(a) and 6(d); *p* < 0.01). These results suggested that EE attenuated ferroptosis by inhibiting the ACSL4 expression.

### 3.5. Enriched Environment Increased the Expression of HIF-1*α* after Cerebral I/R Injury

**Figure 7:**
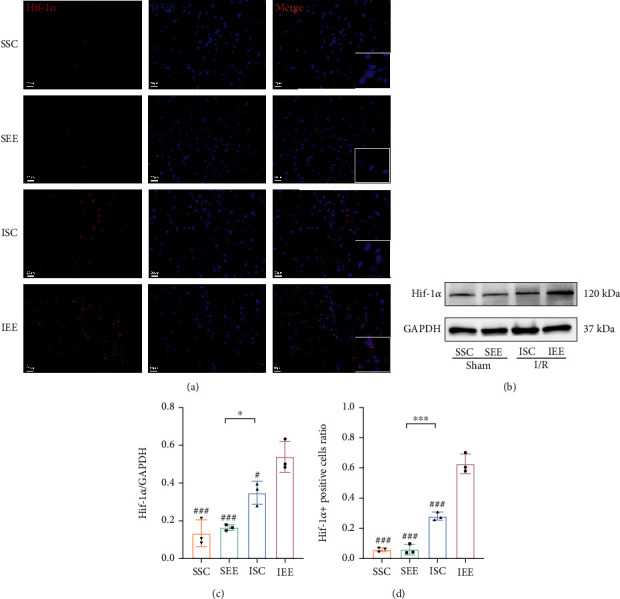
Enriched environment increased the expression of HIF-1*α* after I/R injury. (a, d) Immunofluorescence images of HIF-1*α* and statistical analysis of the positive rate. (b, c) Western blots and quantification of HIF-1*α* in the peri-infarct cortex. Scale bars, 20 *μ*m. *n* = 3. Data are expressed as mean ± SD. ^∗^*p* < 0.05 and ^∗∗∗^*p* < 0.001 vs. SEE group; ^#^*p* < 0.05 and ^###^*p* < 0.001 vs. IEE group.

As HIF-1*α* was reported as the transcriptional regulator of ACSL4, we further investigated whether EE attenuated ferroptosis via the HIF-1*α*-ACSL4 pathway. Western blot results revealed that the HIF-1*α* expression level of the I/R groups obviously increased versus the sham-operated groups. Moreover, HIF-1*α* expression levels were uplifted in the IEE group versus the ISC group (Figures 7(b) and 7(c); *p* < 0.05). These results were further supported by immunofluorescence results. In addition, HIF-1*α* was mostly expressed in the nucleus as it was colocalized with DAPI, which indicated that HIF-1*α* might act as a transcription factor (Figures 7(a) and 7(d); *p* < 0.001). To clarify the underlying mechanism of how HIF-1*α* negatively regulated ACSL4 expression, we further used qRT-PCR to examine the ACSL4 mRNA level. The outcomes demonstrated that the ACSL4 mRNA level of the IEE group was considerably less than the ISC group, which suggested that HIF-1*α* might inhibit ACSL4 expression at the transcriptional level (Figure 6(e); *p* < 0.05).

## 4. Discussion

While stroke mortality is declining because of advances in thrombectomy and thrombolytic therapies, disability caused by IS remains high [41, 42]. Disability or functional impairment after IS severely affects the life quality of survivors and imposes a heavy burden on their families [12]. It is a top priority to find ways to reduce functional deficits after IS. Growing evidence in animal models has shown that EE can promote functional recovery after IS [43, 44]. Exploring the mechanisms underlying EE will help to optimize EE setting, potentially benefiting clinical practice in the future.

To test the effect of EE on neurological function, a series of behavioral experiments were conducted. The results of mNSS suggested that neurological function was impaired in I/R rats while EE reversed the damage. The mNSS included motor, sensory, reflex, and balance assessments. In this study, the results of mNSS indicated that EE could reverse motor dysfunction, sensory abnormalities, and balance disorders caused by I/R injury, thereby improving neurobehavioral function [45]. The results of MWM indicated that EE improved long-term spatial learning and memory functions after stroke. Previous studies have also shown that EE can reduce neurological deficits after I/R injuries, thereby reducing mNSS scores [10, 46]. These results were consistent with our study.

Ferroptosis was closely associated with neurological diseases. The accumulation of excess iron in intracerebral hemorrhage (ICH) could lead to oxidative stress and neuronal damage. Inhibiting ferroptosis by regulating the miR-124/ferroportin signaling pathway could ameliorate neuronal cell death after ICH [47]. In ischemic stroke, lipid peroxidation increased while GSH expression decreased in the injured region, and inhibiting this change mediated a neuroprotective effect [48]. Iron chelators, such as ferrostatin-1 (Fer-1) and liproxstatin-1 (Lip-1) that act as ferroptosis inhibitors, could protect the neurovascular unit in acute stroke [49]. However, the precise mechanism by which ferroptosis was involved in the pathophysiological process of stroke is unclear. Previous studies have shown that exercise training could improve functional recovery after stroke by inhibiting ferroptosis [50]. However, there was still no evidence whether EE-mediated neurological recovery was associated with ferroptosis. We examined key indicators of ferroptosis to investigate whether EE-mediated neuroprotection was mediated by modulating ferroptosis. The TUNEL assay can detect and quantify cells undergoing regulated cell death, including ferroptosis, by labeling indicative of endonucleolytic cleavage of DNA [51]. According to the TUNEL assay results, EE significantly inhibited regulated cell death. GPX4 scavenges lipid hydroperoxides to prevent ferroptosis [52]. The GPX4 expression was then examined in different groups, and EE significantly improved the level of GPX4 after I/R injury. One feature of ferroptosis is iron accumulation [53]. To investigate the changes in iron after cerebral I/R injury and whether EE could alter iron content, we used Perls' staining and iron assay kit to detect iron alterations. The results showed that cerebral I/R injury obviously increased the iron level, whereas EE could decrease the iron level. Another feature of ferroptosis is lipid peroxidation due to iron accumulation [54]. Lipid peroxidation generates a variety of oxidation products, MDA being one of the most common [55]. The MDA assay results revealed that changes in MDA were consistent with the changes in iron content. We hypothesize that EE may reduce ferroptosis and thus promote recovery after cerebral I/R injury based on these alterations in ferroptosis-related factors.

Given that ferroptosis may be regulated by inflammatory cytokines, the inflammatory cytokines (TNF*α*, IL-6, and IL-1*β*) and mRNA expression levels were measured using qRT-PCR. The results showed that EE reduced the mRNA expression levels of inflammatory cytokines after I/R injury indicating that EE may attenuate ferroptosis by inhibiting neuroinflammation. Our findings were supported by several studies. An earlier study has demonstrated that the increased expression of IL-1*β* made cells more susceptible to ferroptosis [56]. Additionally, IL-6 may potentially interfere with iron homeostasis and induce ferroptosis [57]. Most importantly, these inflammatory cytokines have been proven to directly affect the GPX4 expression level, indicating that inflammatory cytokines may be able to regulate ferroptosis [58]. However, numerous studies have shown that the presence of ferroptosis could also affect the expression of inflammatory factors [58, 59]. Ferroptosis played an important role in the model of nonalcoholic steatohepatitis, since it served as the trigger for inflammation [60]. In the model of psoriatic, inhibition of ferroptosis reduced the production of cytokines including TNF*α*, IL-6, and IL-1*β* [61]. In the model of diabetes, ferroptosis induced inflammation in the diabetic wound, and the application of ferroptosis inhibitors reduced the expression of inflammation markers [62]. More research is required to determine how inflammation and ferroptosis are related. Further research is needed to determine the precise involvement of EE in the interplay between ferroptosis and the inflammatory response following stroke.

Our previous studies have identified some mechanisms of EE on functional recovery after cerebral I/R injury, including the promotion of vascular regeneration and inhibition of neuronal apoptosis and pyroptosis [9, 13, 34]. This study also showed that EE could promote the expression of HIF-1*α*. HIF-1*α* could regulate many target genes as a transcription factor [29, 63]. Vascular endothelial growth factor (VEGF) is one of the downstream target genes of HIF-1*α*, which is extensively involved in the pathological process of ischemic stroke [64, 65]. HIF-1*α* plays a neuroprotective role by regulating VEGF-mediated angiogenesis and neuroregeneration [66]. In addition, HIF-1*α* has a neuroprotective effect by increasing erythropoietin expression, which can enhance oxygen transport and increase cerebral blood flow [67, 68]. However, several studies have also pointed out that in the early stages of ischemic stroke, HIF-1*α* aggravates neurological damage by exacerbating the permeability of the blood-brain barrier and mediating the expression of proinflammatory factors and inflammatory responses [69–71]. The same molecule may play very different roles at various stages of the disease pathology. Therefore, it is necessary to further investigate the complex and significant role that HIF-1*α* plays in ischemic stroke. According to the western blot results, cerebral I/R injury markedly increased the expression level of HIF-1*α*, while EE could further increase the HIF-1*α* expression. Immunofluorescence results also showed that EE increased the expression of HIF-1*α* mainly in the nucleus which further supported the idea that HIF-1*α* might act as a transcription factor. These results implied that HIF-1*α* played a fundamental role in the neuroprotective effect of EE after cerebral I/R injury.

Increased expression of ACSL4 exacerbates brain injury by making cells more susceptible to ROS-induced ferroptosis [26, 27, 28, 72]. In our study, western blot results showed that cerebral I/R injury remarkably elevated the expression of ACSL4, while EE reduced the expression of ACSL4. Double immunofluorescence staining showed that ACSL4 was predominantly expressed in neurons after cerebral I/R injury, and EE reduced the proportion of ACSL4/Neun-positive cells. We hypothesized that EE could enhance neuronal tolerance to ROS caused by cerebral I/R injury by decreasing ACSL4 expression. As a lipid metabolism enzyme, ACSL4 is necessary for ferroptosis, which results in increased lipid peroxidation and ferroptosis [73]. Recently, ACSL4 has already been regarded as a ferroptosis modulators [23]. Numerous studies have demonstrated that increased ACSL4 expression can promote ferroptosis and the inhibition of ACSL4 expression could reduce ferroptosis [74]. Decreased ferroptosis is inextricably linked to decreased ACSL4 expression, and when ferroptosis inducers are applied, ACSL4 expression is elevated [75]. In future studies, we should involve overexpressing ACSL4 to further determine the relationship between EE-suppressed ferroptosis and ACSL4. Besides, the qRT-PCR results indicated that the ACSL4 reduction occurred at the transcriptional level. Recent studies showed that HIF-1*α* was bond to the ACSL4 promoter region to repress its transcription [28, 32]. Our study further verified that the increase in HIF-1*α* expression level induced by EE might also affect the transcription of ACSL4 after cerebral I/R injury. Previous studies confirmed that the ischemia-induced increase in HIF-1*α* expression could suppress ACSL4 expression after oxygen and glucose deprivation (OGD). Knockdown of HIF-1*α* in SH-SY5Y enhanced the expression of ACSL4 following OGD. After OGD treatment, HIF-1*α* binds to the conserved noncoding sequences 1 and 2 promoter regions of ACSL4 thereby suppressing the expression of ACSL4 [28]. It was further established that HIF-1*α* negatively regulates the expression of ACSL4 in vitro. In HK-2 cells, ACSL4 expression increased after the knockdown of HIF-1*α*. The application of HIF-1*α* inhibitors could enhance the mRNA level of ACSL4 [32]. Our experimental results are consistent with previous studies. Less perfection of our study was the lack of HIF-1*α* knockdown experiments to verify the deterministic effect of EE on the expression of HIF-1*α*. Further studies should be conducted on this aspect in the future.

As an important environmental intervention, EE inhibits ferroptosis and improves functional recovery after I/R injury. Our findings showed new light on the potential therapeutic mechanisms of EE as well as the pathophysiological development of stroke recovery. This also provides us with novel insight for poststroke rehabilitation in clinical transformation. For example, clinical patients can be treated in an integrated EE-like environment that includes appropriate intensity physical activity, active social interaction, a mindful natural environment, and appropriate challenging tasks to accelerate recovery after stroke. Also, treatments that target ferroptosis and its related molecules may be a new option for the rehabilitation of stroke. However, there are still some limitations in our study. We only explored the effect of EE on ferroptosis after stroke. The effect of EE on other forms of cell death and their relevance remain to be explored. Also, we only investigated the effect of a specific duration of EE intervention on stroke recovery. The effect of different duration of EE intervention on stroke recovery remains to be investigated. We will work to optimize our experimental protocols and designs in future studies.

## 5. Conclusions

In this study, we confirmed the effectiveness of EE in promoting functional recovery after cerebral I/R injury by attenuating ferroptosis. And this process might be activated by the HIF-1*α*-ACSL4 pathway. EE also inhibited the expression levels of inflammatory cytokines including TNF*α*, IL-6, and IL-1*β*, which might facilitate functional recovery after stroke. This study provides more theoretical evidence that EE is a promising rehabilitation strategy for stroke.

## Data Availability

The article contains the original contributions discussed in the study; further questions should be addressed to the relevant author(s).
